# Hatchability of *Fascioloides magna* Eggs in Cervids

**DOI:** 10.3390/pathogens12050741

**Published:** 2023-05-22

**Authors:** Tibor Halász, Tamás Tari, Eszter Nagy, Gábor Nagy, Ágnes Csivincsik

**Affiliations:** 1 Institute of Physiology and Animal Nutrition, Kaposvár Campus, Hungarian University of Agriculture and Life Sciences, H-7400 Kaposvár, Hungary; halasz.tibor@sefag.hu (T.H.); csivincsik.agnes@uni-mate.hu (Á.C.); 2 Zselic Wildlife Estate, Somogy County Forest Management and Wood Industry Share Co., Ltd., H-7400 Kaposvár, Hungary; 3 Institute of Wildlife Management and Wildlife Biology, Faculty of Forestry, University of Sopron, H-9400 Sopron, Hungary; tari.tamas@uni-sopron.hu (T.T.); nagy.gesztenye07@gmail.com (E.N.); 4 One Health Working Group, Kaposvár Campus, Hungarian University of Agriculture and Life Sciences, H-7400 Kaposvár, Hungary

**Keywords:** roe deer, red deer, *Fascioloides magna*, egg hatching

## Abstract

The giant liver fluke (*Fascioloides magna*) is an invasive parasite found permanently in three foci in Europe. The fluke has an indirect life cycle involving a final and an intermediate host. The currently accepted terminology determines three types of final hosts: definitive, dead-end, and aberrant hosts. Recently, roe deer (*Capreolus capreolus*) has been classified as an aberrant host, which cannot contribute to the reproduction of *F. magna*. This study investigated the hatchability of *F. magna* eggs of red deer (*Cervus elaphus*) and roe deer origin to compare the suitability of the two host species for the maintenance of the parasite. The study was carried out on a newly invaded area, two years after the first reported observation of *F. magna*. The prevalence of the parasite proved to be 68.4% (CI95% 44.6–85.3%) in red deer and 36.7% (CI95% 24.8–50.0%) in roe deer. The difference between the two species was confirmed to be significant (*p* = 0.02). The mean intensity proved to be 10.0 (CI95% 4.9–22.6) and 7.59 (CI95% 2.7–24.2) in the red deer and the roe deer, respectively. The difference of the mean intensities did not prove to be significant (*p* = 0.72). Of the 70 observed pseudocysts, 67 originated from red deer and 3 from roe deer. Most of the pseudocysts contained two flukes, while a few pseudocysts contained one or three parasites. Egg production was observed in all three types of pseudocysts. We did not find more than three flukes in any pseudocyst. The apparent proportion of self-fertilisation in flukes without mating partners was 23.5% and 100% in red deer and roe deer, respectively. The survival of single-parent eggs was not confirmed to be worse than that of gregarious parents. The viability of offspring originating from roe and red deer differed significantly. Our findings suggest that *F. magna* adapted to the new populations of susceptible hosts rather than vice versa.

## 1. Introduction

The giant liver fluke (*Fascioloides magna*), also known as large American liver fluke, is an invasive parasite found permanently on the northern part of the American continent and in three foci in Europe [1,2]. The fluke has an indirect life cycle involving a final and an intermediate host. In this complex life cycle, mainly pulmonate freshwater snails belonging to the family Limnaeidae can serve as the most common intermediate host [3]. According to the currently accepted terminology specified by Pybus [1], there are three basic final host categories: (1) definitive host, e.g., white-tailed deer (*Odocoileus virginianus*), mule deer (*Odocoileus hemionus*), red deer (*Cervus elaphus*), fallow deer (*Damad ama*); (2) dead-end host, e.g., moose (*Alces alces*), cattle, wild boar (*Sus scrofa*); and (3) aberrant host, e.g., roe deer (*Capreolus capreolus*), mouflon (*Ovis aries musimon*), sheep, and goat [2]. The definitive host is the most uniformly defined in the literature. In these species, the giant liver flukes become adults in a thick wall pseudocyst located within the liver parenchyma. They produce viable eggs, which can pass through into the small intestine and are shed by faecal material into the external environment. These hosts contribute crucially to the spread of the parasite. In dead-end hosts, the flukes can successfully reach the liver but rarely mature. Only a few eggs are produced, which remain enclosed in the liver parenchyma. The infection in definitive hosts and dead-end hosts rarely have a deadly effect. The giant liver fluke causes the most serious liver damage in aberrant hosts and often results in the death of this type of host. The general supposition is that the parasite cannot accomplish its migration and maturation inside the host body. The immature flukes may reach the liver, but the pseudocyst formation and egg production are usually unsuccessful. Contrary to the definitive host, dead-end hosts and aberrant hosts do not contribute to the spread of the parasite [1,2,3,4].

Aside from some wild ruminants, e.g., mouflon and chamois (*Rupicapra rupicapra*), the roe deer is the only cervid species in Europe known as an aberrant host. Despite the aforementioned aberrant host characterisation, in recent years, some studies revealed that pseudocyst formation, fluke maturation, and egg production are not uncommon phenomena as previously described. In a Croatian survey, 227 liver and faecal samples were analysed to assess the presence of *F. magna* in differently located roe deer populations [5]. Fourteen of the organs contained active pseudocysts. Seven animals had both pseudocysts containing sexually mature flukes and migratory juvenile flukes simultaneously. These findings suggested the longer survival of the large American liver fluke-infected roe deer. Despite adult parasites, none of the faecal samples was positive for the *F. magna* egg. The authors concluded that the observed phenomena indicated a potential beginning of adaptation processes in roe deer [5].

Fifty-two faecal samples were analysed to assess the prevalence and epidemiological risk of *F. magna* infection in roe deer [6]. The samples were collected in 2015 (n = 35) and 2017 (n = 17) in the Lower Silesian Wilderness, Poland. The prevalence of shed eggs was 45.7% and 29.4% in the first and second study years, respectively. These results demonstrated, on the one hand, that roe deer also could have a considerable role in the deposition of large American liver fluke eggs into the environment. On the other hand, the presence of the eggs in faecal material could indicate a co-evolutionary host–parasite relationship [6].

This investigation aimed to determine the hatchability of giant American liver fluke eggs originating from roe deer in comparison with red deer-originated eggs to demonstrate the co-evolutionary process between the parasite and its new host.

## 2. Material and Methods

### 2.1. Sample Collection

We collected the samples from a regional endemic area in the Southern Transdanubian region of Hungary, where the presence of the giant liver fluke was confirmed in 2016, two years before the initiation of this investigation [7]. The study was conducted between 1 January 2018 and 31 December 2022 on a hunting area managed by SEFAG Forest Management and Wood Industry Share Company.

For the hatching investigation, we collected *F. magna*-infected roe deer and red deer livers. Every animal was shot in the frame of individual hunting events and not for the aim of our study. After the deer were gralloched, we separated the liver and immediately transported them to the laboratory.

### 2.2. Parasitological Procedure

We kept the organs at 4 °C and processed them in 24 h. We sliced the organs into 0.5–1 cm wide segments during the liver autopsy to determine the pseudocyst numbers and their fluke counts. After a cyst opened, its content was immediately gathered using a syringe and washed through a sieve with 100 µM diameter pores. For hatching, the eggs were collected from both host species. We collected the one-fluked pseudocysts, the two-fluked pseudocysts, and the three-fluked pseudocysts separately. After this procedure, we counted the fluke number and thoroughly washed the cutting surface off to avoid egg contamination.

### 2.3. Histopathology

For microscopical investigation, the pseudocyst containing roe deer livers and some tissue blocks were taken from the infected organs. The sample blocks were fixed in 10% buffered formaline. Before the haematoxylin–eosin staining, the samples were embedded into paraffin and cut into four μm thick sections.

### 2.4. Hatching Procedure

**Figure 1:**
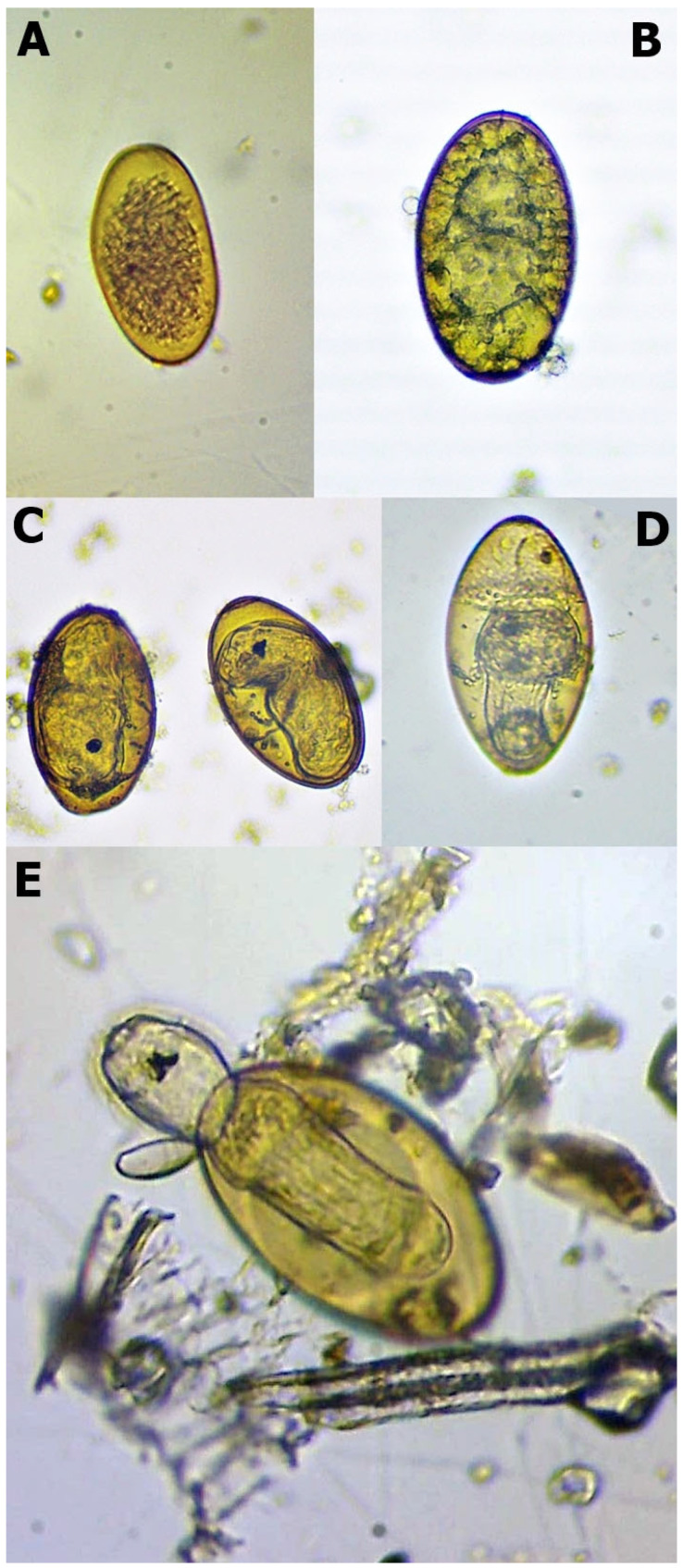
Different stages of *Fascioloides magna* eggs during the hatching (note: (**A**)—unembryonated egg; (**B**)—embryonated egg; (**C**)—eye-spotted miracidium; (**D**)—pre-hatching miracidium, and (**E**)—hatching miracidium).

The eggs were placed into six-well plates filled with distilled water (approximately 10 mL) and were incubated at 25 °C. The evaporated water was continuously checked and refilled. The hatching procedure lasted for six weeks. After the tenth day, we checked the embryonation. In this phase, the development generally cannot be detected. We assessed the eggs weekly during the remaining time and used Swales’ [8] and Campbell’s [9] works for their developmental evaluation. We divided the developmental stages into five categories (Table 1, Figure 1). Before the hatching process, the final amounts of different originated eggs were divided into three portions. Therefore, the final results of each isolate combined from three repetitions.

The hatching of *F. magna* miracidia intermittently occurs. The temperature decrease could stimulate and accelerate the process [8,9]. For this reason, we placed the pre-hatching miracidium stage eggs into the refrigerator at 4 °C for two h. After this period, the eggs were stored at room temperature for 60 min, and the hatching process was assessed using a microscope with 40× magnification. The cooling and observational periods would have been repeated if the miracidium had not emerged. After two resultless attempts, we qualified the hatching to be unsuccessful.

### 2.5. Statistical Analysis

Characterising the fluke infection in hosts, we determined the prevalence and mean intensity in them. For comparing the results, we used unconditional exact test [10]. The proportions of hatched, pre-hatching, eye-spotted miracidia, and embryonated eggs were determined. To compare the hatching ability of one-fluked and two-fluked eggs originating from the two investigated host species, we conducted a Kaplan–Meier survival analysis. For this reason, we classified the eggs into the following groups: red deer one-fluked pseudocyst egg (RED-PC1), red deer two-fluked pseudocyst egg (RED-PC2), roe deer one-fluked pseudocyst egg (ROE-PC1), and roe deer two-fluked pseudocyst egg (ROE-PC2). The differences of the cumulative survival curves were ascertained by log-rank test. The statistical analysis was performed by SPSS statistical software, version 27.0 [11].

## 3. Results

### 3.1. Parasitological Findings

Our investigation found 13 and 22 *F. magna* infected animals among 19 red deer and 60 roe deer, respectively. The prevalence proved 68.4% (CI95% 44.6–85.3%) in red deer and 36.7% (CI95% 24.8–50.0%) in roe deer. The difference between the two species was confirmed to be significant (*p* = 0.02). The mean intensity proved 10.0 (CI95% 4.9–22.6) and 7.59 (CI95% 2.7–24.2) in the red deer and the roe deer, respectively. The difference of the mean intensities did not prove to be significant (*p* = 0.72). Of the 70 observed pseudocysts, 67 originated from red deer and 3 from roe deer. The main prevalent pseudocysts contained two flukes, while one or three parasites were found in smaller amounts. Egg production was observed in all three types of pseudocysts (Table 2). We did not find more than three flukes in any pseudocyst.

### 3.2. Histopathology

**Figure 2:**
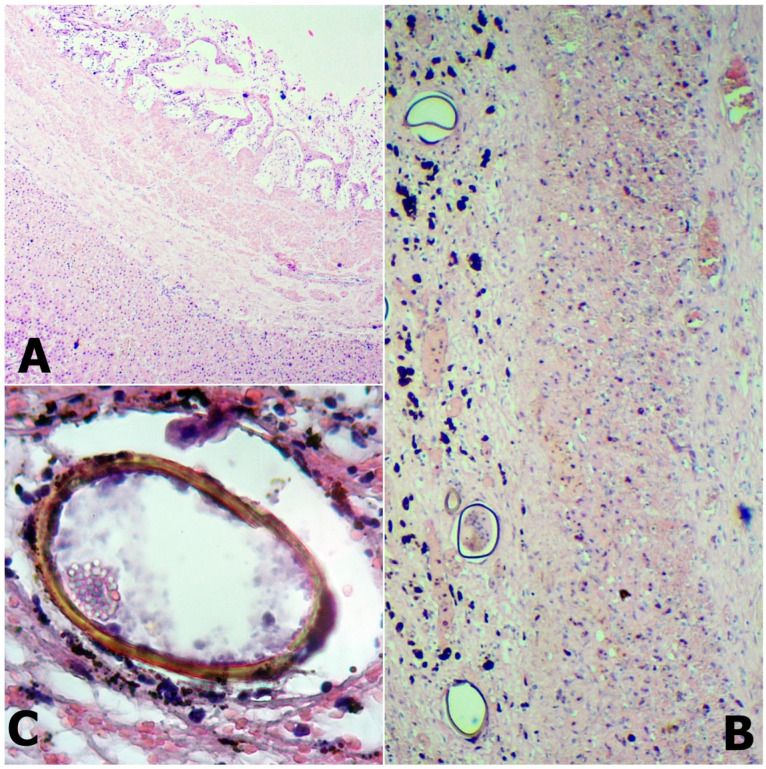
The fibrous cyst wall (**A**) comprises thick connective tissue and diffused inflammatory cells. Inside the cyst lumen (**B**), egg aggregation can be observed. *Fascioloides magna* egg in roe deer liver (**C**).

In three infected roe deer livers, we observed the formation of thick-walled pseudocysts. These organs had partly or completely disrupted texture due to the extensive interstitial fibrous connective tissue proliferation. Due to the inflammatory changes, the proliferated connective tissue stripes were formed, wherein thick-walled hepatic blood vessels and intrahepatic bile ducts were observed. At the cutting surface, we observed the migratory tract of the parasites, which usually contained dark brown pigmentation, the haematin. The cysts in the liver parenchyma consisted of thickened connective tissue, infiltrating lymphocytes, and eosinophil granulocytes. Several eggs were situated in the lumen (Figure 2).

### 3.3. Egg Hatching

We collected 1080 eggs altogether (307 from RED-PC1, 402 from RED-PC2, 99 from ROE-PC1, and 278 from ROE-PC2) for hatching (Table 3).

**Figure 3:**
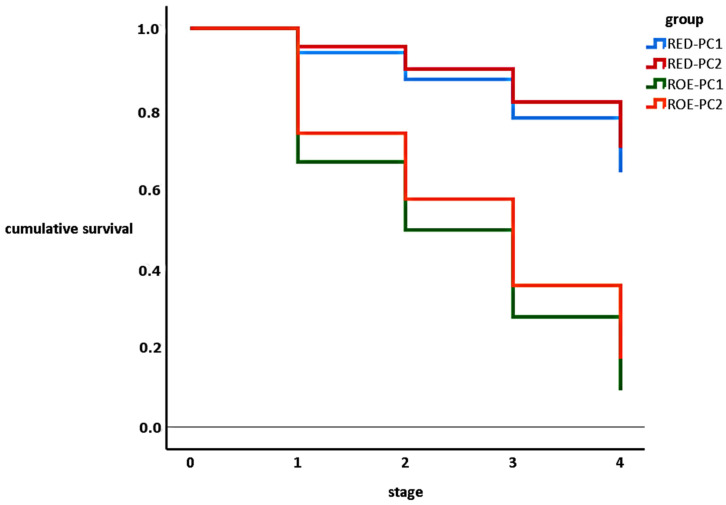
Kaplan–Meier survival curves of *F. magna* eggs originated from different types of hosts and pseudocysts. (Note: stage 0: fresh, 1: embryonated, 2: eye-spotted, 3, pre-hatching, 4: hatching).

The more considerable developmental retardation was observed between the fresh (one-cell stage) and embryonated phases in both hosts. After embryonation, the developmental losses were moderate until hatching. Interestingly, we found that all eye-spotted eggs reached the pre-hatching stage despite the host or the fluke number (Figure 3.).

In the comparison of the development within the hosts, we found strong divergences between the different egg origins (viz. RED-PC1 vs. ROE-PC1 and RED-PC2 vs. ROE-PC2). The hatching success did not vary between the one-fluked and two-fluked pseudocysts, neither in red deer nor roe deer (Table 4).

## 4. Discussion

Our results supported a previous finding. The *F. magna* could be able to reach sexual maturity and produce fertile eggs in roe deer [6], whose species is currently known as the aberrant host. On the other hand, we confirmed that the parasite has the potential for selfing (self-fertilisation), which was previously observed in its relatives, within the order Echinostomida [12,13].

Giant liver fluke is apparently an invasive parasite in Europe and this species is capable to establish new host populations and spread rapidly between new habitats. This invasive parasite may alter the population dynamics of different types of final hosts [14]. As an alien parasite, its co-existence with roe deer has lasted for a short period; therefore, the co-evolutionary adaption of the new host has only recently begun [5]. The signs of this possible co-evolutionary process can be detected. Within its older European habitats, faecal egg shedding is also confirmed [6], while it is lacking in newly invaded areas [5]. Pseudocyst formation in roe deer, as the sign of an advanced stage of infection, has already been found in all European *F. magna* habitats [15].

During this investigation, we confirmed that large American liver flukes are capable of self-fertilisation in both red deer and roe deer hosts. This phenomenon is characteristic for these species, which have a low probability of meeting a partner because of low mobility or population density. During the colonisation of a new host, at the initial stage, the parasite must cope with low population density. Under these conditions, selfing ensures reproduction, thus maintaining the genotype until a potential mating partner appears [14].

As a consequence of self-fertilisation, the population loses genetic diversity through inbreeding [15]. In liver fluke (*Fasciola hepatica*) without co-inhabitants, self-fertilisation occurs in only 2% [16], which suggests that this reproductive strategy is a facultative way of population maintenance.

In our study, the apparent proportion of selfing in flukes without mating partners was 23.5% and 100% in red deer and roe deer, respectively. Our data suggested that parasites in roe deer have a much higher selfing ability. The very few (only one) single mature flukes in roe deer might explain this controversy.

The limited efficacy of self-fertilisation was conspicuous when we investigated the average loss during embryonic development. In comparison with eggs originating from gregarious flukes, single-parent eggs faced heavier losses, even though the difference between the viability of cross-fertilised and self-fertilised eggs did not prove significant.

The difference between the two host species was remarkable. The eggs of red deer origin showed a significantly better survival potential than that of roe deer origin. Based on this observation, the roe deer host seemed to impede the development of the parasite more efficiently than the red deer. The findings of other studies cannot support this hypothesis [4]. It is more probable that our sampling method (shooting healthy animals) could detect only the fortunate survivors of the parasite infection. These animals could acquire less virulent genotypes of *F. magna*.

Within the study area, *F. magna* is a new parasite for the local populations of both the red and roe deer [7]. Considering the prevalence and the mean intensity we detected in the two investigated species, the parasite could establish a permanent population in the region. The lower prevalence in the roe deer cannot be explained by higher resistance. The survival of a roe deer individual depends on the environmental factors of its territory. The presence or absence of other definitive hosts and intermediate hosts strongly influence the probability of infection. For this reason, the comparison between the two species is less productive than a comparison of roe deer populations of different areas.

In new areas with new host species or populations, a parasite must choose an optimal level of virulence to maximise its reproduction success [17,18]. In this situation, high virulence reduces the host’s fitness as well as its lifespan, which threaten the survival of the parasite and can drive the extinction of the whole parasite population. The reduction in virulence in favour of better transmissibility can be observed in many parasite species [17,18,19,20].

In the case of *F. magna*, it is better to consider host–parasite interactions, whereas this parasite has no intention to kill the host. Since its first detection within the study site, two years had passed until the beginning of this investigation [7]. These circumstances suggested that the parasite had resided for a very short period, which could not be enough for a population level genetic change in deer populations of the area. On the other hand, the parasite could have produced several generations, even during this short period of time. Moreover, the loss by perished hosts could cause a bottle-neck effect in the parasite population. Highly virulent genotypes, which caused the death of their hosts, reached a dead end without reproduction. Meanwhile, less virulent genotypes could multiply the locally advantageous alleles, causing radical change in gene frequency in parasite population.

Since its occurrence, the *F. magna* could not cause an observable population reduction in any of its host populations in the study region. A stable equilibrium between the host and the parasite seemed to have evolved at the very beginning of the parasite invasion. Notwithstanding, by a more reliable explanation, the parasite and the host continue a balanced trench warfare, in which both participants have victories and defeats time after time. This situation is created by a fluctuating selection in both the parasite and the host caused by themselves to each other as the Red Queen hypothesis describes it expressively [21,22].

This balanced arms race suggests that a heterogenous host population met a less heterogenous parasite population, which can prevent the escalation of virulence [23,24]. Without genetic investigations, this hypothesis is rather speculative; therefore, further research is needed to clarify the background of the surprisingly attentive behaviour of the invader in the study area.

This study aimed to investigate an aspect of the roe deer and *F. magna* co-evolution. In a hatchability test, we confirmed that eggs obtained from the pseudocysts of roe deer livers can develop and hatch. This finding supported the hypothesis that the roe deer may serve as a definitive host for *F. magna* after a short adaptation period. Notwithstanding, in this study, the adaptation period was as short as two years, which questioned the adaption of the host. Based on our findings, it is more probable that the parasite sacrificed its virulence in favour of transmissibility.

## Figures and Tables

**Table 1:** Stages to assess the development of *Fascioloides magna* eggs in roe deer samples.

Developmental Category	Main Characteristics
Unembryonated egg	No developmental processing, the eggs usually seem empty or damaged (Figure 1A)
Embryonated egg	Embryo position in the egg’s centre, embryo formation visible (Figure 1B)
Eye-spot development	Movement often observable eye-spots appeared (Figure 1C)
Pre-hatching miracidium	Intensive movement increased mucoid plug (Figure 1D)
Hatching	Opened operculum miracidia outside the egg (Figure 1E)

**Table 2:** *Fascioloides magna* numbers in the detected pseudocysts.

	One Fluke	Two Flukes	Three Flukes
red deer	17 (4) *	46 (45)	7 (7)
roe deer	1 (1)	2 (1)	0

* The parenthetical numbers indicate the egg containing pseudocysts.

**Table 3:** Percentage of the different developmental stages of one-fluked and two-fluked pseudocysts in both hosts.

	Total Egg	Embryonated	Eye-Spotted	Pre-Hatching	Hatching
RED-PC1	212	80.2% (170) *	64.2% (136)	64.2% (136)	52.8% (1112)
RED-PC2	232	84.5% (196)	70.3% (163)	70.3% (163)	60.3% (140)
ROE-PC1	83	36.9% (31)	14.3% (12)	14.3% (12)	4.8% (4)
ROE-PC2	278	44.8% (60)	20.1% (27)	20.1% (27)	9.7% (13)

RED-PC1: Red deer one-fluked pseudocyst, RED-PC2: red deer two-fluked pseudocyst, ROE-PC1: roe deer one-fluked pseudocyst, ROE-PC2: roe deer two-fluked pseudocyst. * Percentage of the developmental category to the total egg count (number of eggs).

**Table 4:** Comparison of the hatching ability of different egg types.

	RED-PC2	ROE-PC1	ROE-PC2
RED-PC1 *	0.059 **	<0.0001	<0.0001
RED-PC2		<0.0001	<0.0001
ROE-PC1			0.056

* RED-PC1: Red deer one-fluked pseudocyst, RED-PC2: red deer two-fluked pseudocyst, ROE-PC1: roe deer one-fluked pseudocyst, ROE-PC2: roe deer two-fluked pseudocyst. ** *p*-value.

## Data Availability

The data presented in this study are available on request from the corresponding author.
